# Cyclodextrin-Based Nanocarriers for 5-Fluorouracil: Cholesteryl Modification Enhances Antitumor Activity Against Colorectal Cancer Cells

**DOI:** 10.2147/IJN.S583792

**Published:** 2026-06-05

**Authors:** Beata Skonieczna, Bartosz Maliszewski, Natalia Wasiluk, Halina Car, Agnieszka Z Wilczewska, Paweł Misiak, Katarzyna Niemirowicz-Laskowska

**Affiliations:** 1 Department of Experimental Pharmacology, Medical University of Bialystok, Bialystok, Poland; 2 Faculty of Chemistry, University of Bialystok, Bialystok, Poland; 3 Doctoral School of the University of Bialystok, University of Bialystok, Bialystok, Poland

**Keywords:** β-cyclodextrin, cholesterol moiety, 5-fluorouracil, colorectal cancer, drug delivery systems

## Abstract

**Background:**

The objective of this study is to evaluate the biological activity of cholesterol-modified cyclodextrin (CD21chol) complexes with 5-fluorouracil (5-FU) prepared at different molar ratios (1:1, 1:2, and 1:3) and to identify the most potent anticancer formulation.

**Methods:**

The inclusion complexes of CD21chol:5-FU were prepared at different molar ratios (1:1, 1:2, and 1:3) and initially characterized based on their physicochemical properties, such as increased negative surface charge and particle size. Subsequently, the biological activity was investigated in the human colorectal cancer cell line DLD-1 by analyzing cell viability, metabolic activity, membrane integrity, and apoptosis (activation of caspases 3/7, 8, and 9). Cytotoxicity was also measured in non-tumorigenic fibroblasts and cardiomyocytes.

**Results:**

The complexation with 5-FU resulted in a shift toward more negative zeta potential values, with the 1:3 CD21chol:5-FU system showing the greatest change and improved physicochemical stability. Particle size analysis revealed a reduction in hydrodynamic diameter at lower drug ratios (1:1 and 1:2), followed by an increase at the 1:3 ratio. Biologically, the 1:3 complex exerted the strongest cytotoxic effect against DLD-1 cells, reducing metabolic activity and cell viability by over 60%, increasing LDH release, and significantly activating caspases 3/7, 8, and 9, indicating engagement of both intrinsic and extrinsic apoptotic pathways. In contrast, only moderate toxicity was observed in CCD-1079SK fibroblasts and H9c2(2–1) cardiomyocytes.

**Conclusion:**

The results indicate that CD21chol-based delivery systems enhance the anticancer activity of 5-FU. The 1:3 complex appears to be the most promising candidate for further development in colorectal cancer therapy.

## Graphical Abstract

**Figure:**
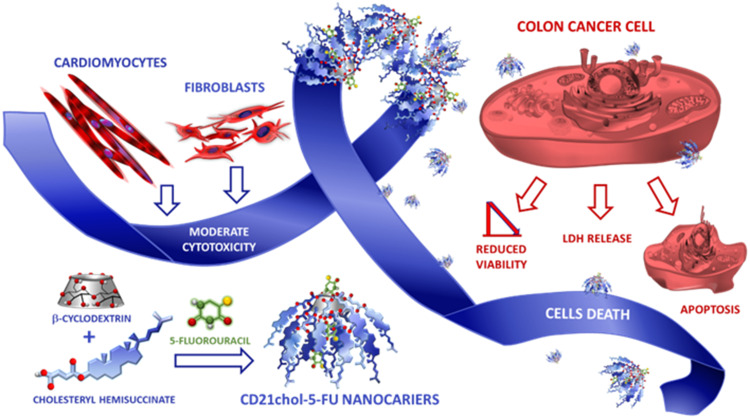

## Introduction

Colorectal cancer (CRC) is the third most frequently diagnosed cancer worldwide. However, it is noteworthy that it ranks second in terms of cancer deaths.^1^ The etiology of the disease is multifactorial, with lifestyle factors representing a primary contributing element. Additionally, genetic and environmental factors have been shown to play a role. As with other diseases, it can be hereditary or non-hereditary. In most cases, it is not inherited but caused by somatic mutations. Colorectal cancer occurs in the colon and rectum. The formation of adenomatous polyps, or adenomas, typically initiates the development of cancer.^2^

As demonstrated by data from 2022, colorectal cancer is among the most prevalent forms of cancer on a global scale and constitutes the second most common cause of cancer-related fatalities. Globally, more than 1.9 million new cases were diagnosed that year, and approximately 904,000 deaths related to the disease were recorded. As early as 2020, colon and rectal cancers were among the most commonly diagnosed cancers, ranking fifth and eighth, respectively, and together accounted for the second leading cause of cancer deaths. Epidemiological analyses consistently show higher incidence and mortality rates among men than among women in all regions of the world. Projections indicate a further increase in the disease burden; by 2050, the number of new cases of colorectal cancer could reach approximately 2.36 million, representing an increase of over 22% compared to 2022.^3^ The DLD-1 cell line is widely used in colorectal cancer research, particularly to elucidate molecular mechanisms, tumor development, and response to anticancer agents. They are employed in both in vitro and in vivo studies in xenograft models to investigate tumorigenesis or the efficacy of anticancer therapy.^4^

The increasing incidence of colorectal cancer is associated with an escalating number of cases resistant to chemotherapy. The efficacy of treatment regimens is progressively diminishing due to the ability of cancer cells to acquire resistance to the treatment, which is responsible for approximately ~90% of the failures of these therapeutic interventions.^5^ 5-Fluorouracil (5-FU) remains one of the cornerstone chemotherapeutic agents in colorectal cancer treatment due to its ability to inhibit Deoxyribonucleic Acid (DNA) synthesis and induce apoptosis in rapidly dividing cells.^6^,^7^ However, several limitations often compromise its clinical effectiveness, including poor aqueous solubility, rapid enzymatic degradation, and systemic toxicity, which lead to severe side effects and limit the achievable therapeutic dose.^8–10^ Moreover, cancer cells develop resistance mechanisms, such as enhanced drug efflux and metabolic inactivation, further diminishing 5-FU efficacy and contributing to treatment failure.^10^ These challenges highlight the urgent need for advanced drug delivery systems to improve the bioavailability, stability, and targeted delivery of 5-FU, thereby enhancing its anticancer activity and reducing adverse effects.^11–14^ Therefore, developing delivery systems that enhance 5-FU selectivity and reduce its systemic side effects remains a key objective in colorectal cancer therapy.

Cyclodextrin-based (CD) carriers are widely recognized for their ability to enhance the therapeutic index of 5-FU by forming stable inclusion complexes, which effectively shield the drug from enzymatic degradation and increase its low aqueous solubility.^15^ The incorporation of lipophilic moieties, such as cholesterol, into the CD framework further optimizes these systems by imparting high affinity for cellular membranes. Interaction with cholesterol-rich lipid rafts not only stabilizes the carrier architecture but also facilitates cellular internalization via endocytosis or transient membrane destabilization, significantly improving the intracellular delivery of the chemotherapeutic payload.^16^,^17^

This study aims to build on previously published results that characterized cholesteryl-modified β-cyclodextrin (CD21chol) as a carrier for 5-fluorouracil.^18^ This approach appears to be a highly promising solution. Cyclodextrin decorated with 21 cholesteryl moieties (CD21chol) retained thermally stable and possessed the ability to form inclusion complexes, making it an excellent candidate for drug delivery applications. In our recent study, we investigated cholesteryl-modified cyclodextrin system as a carrier for hydrophobic anticancer agents such as doxorubicin, demonstrating their ability to form stable inclusion complexes and exhibit enhanced biological activity.^19^ Building on these findings, the present work extends this system toward a structurally distinct, small and highly hydrophilic drug, 5-fluorouracil, whose interaction with cyclodextrin-based carriers remains less well understood. This allows us to evaluate the versatility and limitations of the previously developed delivery platform beyond strongly interacting guest molecules. These complexes facilitate the delivery of therapeutic agents directly to cancer cells, thereby reducing their toxicity to non-targeted cells. To this end, the compatibility and cytotoxic potential of the complexes were examined using various methods. The complexes with 5-fluorouracil were evaluated against representatives of normal cells (CCD-1079Sk fibroblasts and H9c2(2–1) cardiomyocyte cells) and colorectal cancer cells (DLD-1 line). The CD21chol:5-FU displayed robust in vitro antineoplastic efficacy, evidenced by its capacity to diminish the viability and proliferation of cancerous cells by disrupting the plasma membrane and inducing apoptosis, as demonstrated by the analysis of caspase pathways. According to the data presented, the cholesteryl-modified cyclodextrin carrier for 5-FU shows considerable promise as a therapeutic nanocarrier, with the potential to modulate 5-FU activity, reduce dosage requirements, and overcome drug resistance.

## Materials and Methods

### Materials

The following reagents were used for the synthesis of the cholesteryl-modified β-cyclodextrin derivative (CD21chol) form previously described procedure^18^ and the preparation of the inclusion complex with 5-FU: cholesterol (92.5%, Sigma-Aldrich), succinic anhydride (Sigma-Aldrich), 4-dimethylaminopyridine (DMAP, 99%, Thermo Scientific), calcium hydride (CaH_2_, 93%, Acros Organics), hydrochloric acid (HCl, 35–38% p.a., Chempur), sodium chloride (NaCl, 99.9%, Chempur), anhydrous sodium sulfate (Na_2_SO_4_, 99%, Chempur), thionyl chloride (99.7%, Acros Organics), β-cyclodextrin (β-CD, 98%, Acros Organics), 5-fluorouracil (5-FU, ≥98%, Sigma-Aldrich), potassium hydroxide (Chempur), sodium hydrogen carbonate (NaHCO_3_, 98%, Chempur), and dry pyridine (Chempur), which was distilled from KOH prior to use. Dry dichloromethane (DCM, Avantor) was distilled from CaH_2_. All organic solvents were obtained from Avantor Performance Materials, Poland S.A.

### Synthetic Procedures

#### Synthesis of Cholesteryl Derivative of β-CD (CD21chol)

The synthesis of CD21chol was carried out in three steps, as previously reported.^18^ First, cholesterol (5.62 g, 14.6 mmol, 1 eq.) was reacted with succinic anhydride (4.37 g, 43.6 mmol, 3 eq.), and DMAP (1.77 g, 14.6 mmol, 1 eq.) in dry pyridine (100 mL). Cholesteryl hemisuccinate (CHEMS)) was isolated by acid extraction (10% HCl) and then purified by recrystallization in methanol to give white crystals weighing 6.5 g. Next, CHEMS (2 g, 4,1 mmol, 1 eq.) was converted to its acid chloride (CHEMS-Cl) using thionyl chloride (0.36 mL, 4.9 mmol, 1.2 eq.) in dry DCM (20 mL). Finally, CHEMS-Cl (2.08 g, 4 mmol, 25 eq.) was reacted with β-cyclodextrin (0.18 g, 0.16 mmol, 1 eq.) in a pyridine/DCM mixture (7:10 v/v) for 24 hours. The product, CD21chol, was isolated by extraction with DCM and purified by acid-base extraction followed by crystallization in warm ethyl acetate. Its structure was confirmed by Nuclear Magnetic Resonance (NMR) and Infrared (IR) spectroscopy, consistent with literature data.

#### Complexation of 5-Fluorouracil with CD21Chol

CD21chol was dissolved in tetrahydrofuran (THF) to prepare a 50 mg·mL^−1^ solution. Aliquots of 1 mL were distributed into 3 bottles. A 5.7 mg·mL^−1^ solution of 5-fluorouracil in methanol was prepared and added to the CD21chol solutions at carrier-to-drug molar ratios of 1:1, 1:2, and 1:3. THF was added to each mixture to reach a final volume of 3 mL. The mixtures were stirred at room temperature for 24 h. After, the solution was added dropwise to water using a syringe pump (0.1 mL·min^−1^) to separate complexes. Suspensions were stirred for 24 h to evaporate solvents, centrifuged, and dried at 60 °C for 24 h. Detailed characterization of these complexes, including their stoichiometry determined by Differential Scanning Calorimetry (DSC), Thermogravimetric Analysis (TGA), Fourier-Transform Infrared spectroscopy (FTIR), and computational methods, has been reported previously.^18^

#### Dynamic Light Scattering (DLS) and Zeta Potential Measurements

To determine the size distribution and colloidal stability of CD21chol and its complexes with 5-FU (molar ratios 1:1, 1:2, 1:3), dynamic light scattering (DLS) analyses were carried out using a Zetasizer Ultra (Malvern Panalytical Ltd., Malvern, UK) equipped with a multi-angle detection system (MADLS). Samples were prepared by dilution of 2 mg·mL^−1^ stock solutions in DMSO to a final concentration of 100 µg·mL^−1^ in phosphate-buffered saline (PBS, pH 7.4), with the final DMSO content adjusted to 5% (v/v). Before analysis, all samples were filtered through 0.45 µm nylon syringe filters to remove dust and large aggregates. MADLS measurements were performed in DTS0012 cuvettes, and scattering was recorded at three angles: 173°, 90°, and 17° (back, side, and forward scattering, respectively). For each sample, five replicate measurements were performed at each angle. To assess the shape and isotropy of the particles, additional measurements were performed at 173° using polarization filters: five with the horizontal (VV) filter and five with the vertical (VH) filter. Electrophoretic light scattering (ELS) measurements were conducted in DTS1070 cuvettes. For each sample, ten replicate measurements were performed. All measurements were carried out at 25 °C.

### Biological Studies

#### Cell Lines, Culture Conditions, and Seeding Procedure

Cytotoxicity of modified cyclodextrins was determined against normal cell lines, including skin fibroblasts CCD-1079Sk and cardiomyocytes H9c2(2–1), as well as their antitumor potential against the colon cancer cell line DLD-1 obtained from ATTC. Cells were cultured in Eagle’s Minimum Essential Medium (EMEM, ATCC) (for CCD-1079Sk) or in Dulbecco Modified Eagle Medium (DMEM) (for DLD-1 and H9c2(2–1)) supplemented with 10% FBS (Gibco) and 1% antibiotic (Penicillin-Streptomycin, 10,000 U·mL^−1^, Gibco) in an incubator set at 37 °C in a 5% CO_2_ atmosphere. After 24 hours, the cells were washed with warm phosphate-buffered saline (PBS, CORNING) and then trypsinized with warm trypsin-EDTA (0.25%, Gibco) to detach them from the medium before being seeded into 96-well plates.

### MTT Assay

Different techniques were used to evaluate the compatibility and cytotoxicity of the tested carriers. A 3-(4,5-dimethylthiazol-2-yl)-2,5-diphenyltetrazolium bromide (MTT) assay was performed to assess cell proliferation and metabolic activity in the presence of the tested carriers. Normal and tumor cells were treated with carriers (50 µg·mL^−1^), including empty CD21chol and complexes at various molar ratios (CD21chol:5-FU 1:1, 1:2, 1:3), and compared with untreated cells. After 24 hours of incubation at 37 °C in 5% CO_2_, the medium was removed, and the cells were washed with warm PBS (CORNING). Then, 100 µL of MTT solution (5 mg·mL^−1^) was added to each well. The plates were incubated at 37 °C for 30 minutes. The MTT solution was carefully removed from each well, and 100 µL of DMSO was added. The plates were incubated at room temperature for about 5 minutes until a purple coloration appeared. In the final step, absorbance was measured using a Varioskan Lux Thermo Fisher plate reader (570 nm).

### Neutral Red Uptake Assay

In another set of experiments, the viability of cells treated with cyclodextrin-based carriers was examined using the neutral red (NRED) assay. Cells were treated with empty and loaded with 5-FU CD21chol in molar ratios of 1:1, 1:2, and 1:3, using drug delivery systems at a concentration of 50 µg·mL^−1^. The control group was untreated cells. After 24 hours of incubation at 37 °C in 5% CO_2_, 10 μL of neutral red solution was added to each well, and the plates were then incubated at 37 °C in 5% CO_2_ for an additional 2 hours. The medium was then gently removed, followed by the addition of a fixative solution (50 µL per well) and a 5-minute incubation at room temperature. After the fixative solution was removed, the solubilizing solution (90 µL per well) was added. Absorbance was measured at 540 nm using a Varioskan Lux Thermo Fisher plate reader.

### Lactate Dehydrogenase Release Assay

To determine the compounds’ capacity to disrupt cell membranes, an Lactate Dehydrogenase (LDH) assay was conducted. DLD-1 cells were treated at a concentration compared to untreated cells. The DLD-1 cells were incubated with modified cyclodextrins (50 µg·mL^−1^) for 24 hours at 37 °C in 5% CO_2_. Then, 50 µL of medium from each well was transferred to a new, clean 96-well plate, leaving the first two rows empty for reduced form of nicotinamide adenine dinucleotide (NADH) standards. These standards were prepared in accordance with the manufacturer’s instructions. A Master Reaction Mix containing LDH buffer and LDH substrate in a 24:1 ratio (48 µL + 2 µL per well) was prepared, and 50 µL of this mixture was added to each well. The plate was stirred on a shaker and incubated at room temperature for 2–3 minutes, protected from light. Finally, the absorbance at 450 nm was measured using a Varioskan Lux Thermo Fisher plate reader. Results are presented as LDH concentration [nM] based on the standard curve.

### Determination of Apoptosis Induction by Caspase 3/7, 8, and 9 Activity Assays

The activity of caspases 9, 8, and 3/7 was measured to study the intrinsic and extrinsic pathways of apoptosis. DLD-1 cells, seeded in 96-well white plates, were exposed to the tested drug delivery systems: empty CD21chol, CD21chol:5-FU 1:1, CD21chol:5-FU 1:2, CD21chol:5-FU 1:3, at concentrations of 50 µg·mL^−1^. Plates were incubated for 24h at 37 °C and 5% CO_2_. Caspase-Glo^®^ 9 reagent (PROMEGA), Caspase-Glo^®^ 8 reagent (PROMEGA), and Apo-ONE^®^ Caspase-3/7 reagent (PROMEGA) were prepared by dissolving the substrate in buffer according to the manufacturer’s instructions. Then, 100 µL of reagent was added to each well and mixed for 30 seconds (300–500 rpm). The plate was left at room temperature for about 30 minutes, protected from light. Then, luminescence (for caspase 8 and 9) or fluorescence (485ex/530m) was measured using a Varioskan Lux plate reader (Thermo Fisher). The results are presented as relative luminescence units (RLU) and relative fluorescence units (RFU).

### Statistical Analysis

Data represent triplicates and are expressed as the mean ± SEM. Differences were considered significant if p<0.05. GraphPad Prism 10.1.2 (GraphPad Software, La Jolla, CA, USA) was used to analyze data using one-way ANOVA followed by Tukey’s multiple-comparison test to compare more than two groups within an experiment.

## Results

Based on our previous studies on the CD21chol system, it was experimentally demonstrated (using spectroscopic and thermal methods) that a maximum of three 5-fluorouracil molecules can associate with a single cyclodextrin derivative.^18^ Even in the presence of a large excess of drug (4–10 equivalents), the system consistently formed 1:3 host–guest complexes. This stoichiometry was further confirmed by quantum-chemical calculations, which indicated that one 5-FU molecule is preferentially located inside the cyclodextrin cavity, while the remaining two interact with the cholesteryl shell. The good agreement between experimental and computational results supports the proposed binding model and provides a consistent framework for interpreting supramolecular organization and drug loading behavior. The observed increase in hydrodynamic diameter at higher drug ratios (1:3) is therefore attributed to saturation of binding sites and possible secondary assembly formation rather than uncontrolled aggregation.

### Zeta Potential of CD21chol:5-FU and Its Implications for Biological Activity

Zeta potential (ζ) measurements were carried out to assess the surface charge of the particles, which plays a crucial role in their colloidal stability, interactions with cell membranes, and mechanisms of cellular internalization.

The cholesterol-bearing cyclodextrin (CD21chol) exhibited a slightly positive zeta potential (+2.95 mV) and a high RSD (81%), indicating heterogeneity and low stability in aqueous media. Upon complexation with 5-FU, a notable shift toward negative values was observed. The zeta potential values for 1:1, 1:2, and 1:3 molar ratios of CD21chol:5-FU were −7.82 mV, −7.84 mV, and −12.35 mV, respectively (Table 1). This progressive decrease suggests drug loading and a modification of the surface character of the complexes toward a more negatively charged profile. Such changes are typically associated with improved dispersion behaviour. However, in physiologically relevant ionic environments, electrostatic stabilization is partially screened, and therefore cannot solely account for colloidal stability. Stability arises from a balance between van der Waals attraction and electrostatic repulsion, yet for cyclodextrin-based systems bearing bulky hydrophobic (cholesterol-derived) substituents, additional steric and hydration forces must be considered. Recent studies on cyclodextrin nanostructures^20^ and functional polymeric assemblies^21^ have highlighted the importance of such contributions in governing stability under biologically relevant conditions.

**Table 1:** Light Scattering Results of CD21chol and Its Complexes with 5-FU

Sample	MADLS		ELS		
	Hydrodynamic diameter [nm]	PDI	Potential ζ [mV]	RSD [%]	Quality factor
**CD21chol**	83.2 ± 23.8	0.94 ± 0.11	+2.95 ± 2.40	81	0.277
**CD21chol:5-FU (1:1)**	65.5 ± 5.9	0.27 ± 0.06	−7.82 ± 1.63	21	0.958
**CD21chol:5-FU (1:2)**	45.5 ± 4.8	0.39 ± 0.08	−7.84 ± 0.90	12	1.051
**CD21chol:5-FU (1:3)**	113.4 ± 2.5	0.16 ± 0.05	−12.35 ± 2.05	17	1.066

An apparent increase in the Quality Factor with increasing 5-FU content (from 0.277 for CD21chol to 1.066 for the 1:3 complex) further supports improved system homogeneity and physical stability, which may facilitate cellular uptake and promote controlled drug release. Modulation of the zeta potential via 5-FU complexation enhances the physicochemical stability of CD21chol-based systems and significantly affects their biological performance, particularly against cancer cells. These findings support the concept that tuning surface charge is critical in the rational design of effective nanocarrier-based drug delivery systems. These findings are in excellent agreement with theoretical predictions, which indicate that the 1:3 complex exhibits the most favorable geometry and the lowest enthalpy of formation.^18^ The consistency between experimental and computational results reinforces the hypothesis that the 1:3 stoichiometry provides this system’s most stable supramolecular architecture.

Multi-angle dynamic light scattering (MADLS) measurements revealed a gradual decrease in hydrodynamic diameter from 83.2 ± 23.8 nm for uncomplexed CD21chol to 45.5 ± 4.8 nm for the CD21chol:5-FU complex at a 1:2 molar ratio. This reduction in particle size suggests that complexation with 5-FU may promote a more compact and uniform supramolecular organization. This interpretation is supported by a significant reduction in the polydispersity index (PDI) from 0.94 ± 0.11 for CD21chol to 0.27 ± 0.06 and 0.39 ± 0.08 for the 1:1 and 1:2 systems, respectively, indicating improved size uniformity. Interestingly, the 1:3 system exhibited an increased hydrodynamic diameter (113.4 ± 2.5 nm) accompanied by the lowest PDI value (0.16 ± 0.05), suggesting the formation of larger but highly homogeneous supramolecular assemblies. This behavior may reflect structural reorganization at higher drug loadings, potentially associated with saturation of available binding sites and the formation of well-defined higher-order structures rather than uncontrolled aggregation, although partial aggregation cannot be fully excluded.

**Figure 1:**
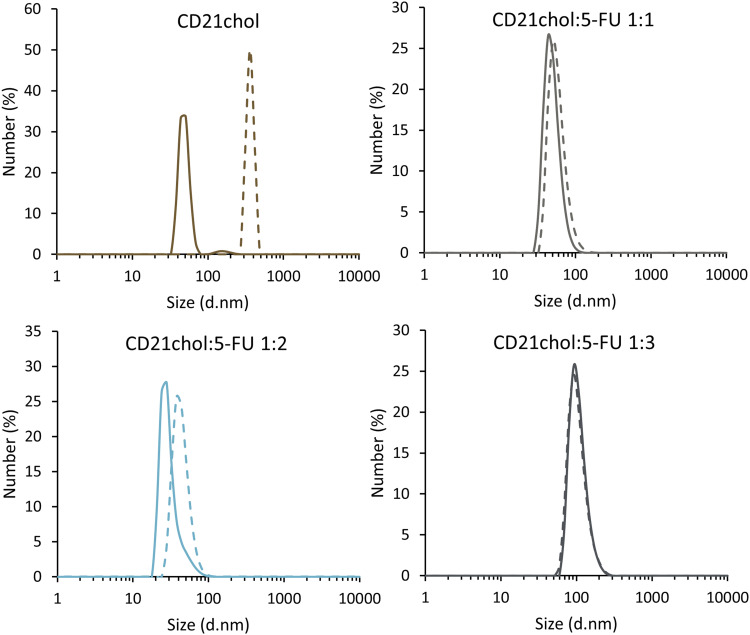
DLS profiles of CD21chol and its complexes with 5-fluorouracil (5-FU) in various molar ratios (1:1, 1:2, 1:3). Solid lines represent measurements obtained using the horizontal polarization filter. In contrast, dashed lines correspond to the vertical filter.

Dynamic light scattering (DLS) measurements were performed using horizontal and vertical polarization filters to separate polarized from depolarized scattering components. This approach allows the detection of deviations from spherical morphology, since non-spherical or anisotropic particles generate a measurable depolarized signal.^22^,^23^ Vertically polarized incident light detected through a vertical filter provides size distribution data largely independent of particle shape in this setup. In contrast, a horizontal filter detects light scattered with orthogonal polarization, which is sensitive to particle anisotropy and deviations from sphericity (Figure 1).

For uncomplexed CD21chol, the scattering profiles revealed a noticeable depolarized component, suggesting the presence of non-spherical particles or anisotropic aggregates. After complexation with 5-FU, the disparity between polarized and depolarized profiles decreased, suggesting a tendency toward a more spherical, uniform particle morphology.

Modifying the zeta potential, alongside improved homogeneity and physicochemical stability, underscores the potential of the CD21chol:5-FU complexes as drug delivery carriers. These changes will likely significantly affect the cellular uptake efficiency of 5-FU in biological environments. Therefore, the next phase of this study focused on biological evaluations to assess the cytotoxicity and anticancer activity of the prepared complexes.

Due to the significant differences in molecular weight and spectroscopic properties between CD21chol and 5-FU, direct experimental investigation of release kinetics was not feasible under standard conditions. However, based on computational insights, a two-step release mechanism may be proposed, involving initial release of 5-FU molecules associated with the cholesteryl moieties followed by slower release of the molecule encapsulated within the cyclodextrin cavity. This hypothesis requires further experimental validation.

### Evaluation of Cytotoxicity of CD21chol:5-FU Against Normal and Colorectal Cancer Cells

To assess whether the observed physicochemical differences translate into biological effects, cytotoxicity assays were performed on cancerous and normal cell lines. In in vitro studies, using normal cells is crucial for assessing the potential impact of therapeutic substances on non-cancerous organs and systems. The most commonly used cells are CCD1079-SK cells derived from human skin fibroblasts. Its primary application is as a control in cellular toxicity studies and in the evaluation of adverse effects associated with novel anticancer pharmaceutical agents.^24^ In turn, cells from the H9c2(2–1) line, derived from rat embryonic heart muscle (strain BD1X), are also frequently used. H9c2(2–1) cells aid in investigating the role of reactive oxygen species and mitochondrial dysfunction in heart disease. It is important to note that such pathology can develop as a result of systemic chemotherapy.^25^ Tests on the above-cited cell lines are typically carried out simultaneously to evaluate the safety profile and efficacy of potential new drugs and drug delivery systems.^26^

**Figure 2:**
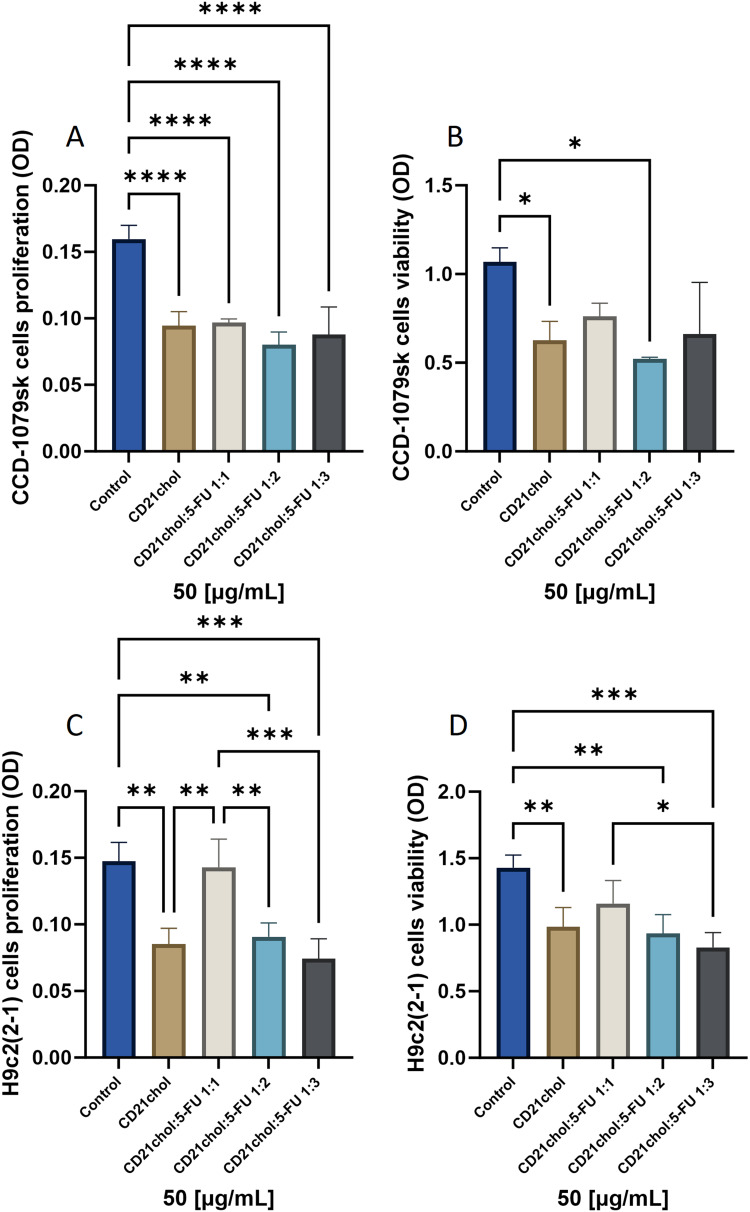
Evaluation of cytotoxicity of CD21chol and its complexes with 5-FU against human fibroblast cells (CCD-1079Sk) and cardiomyocytes (H9c2(2–1)). (**A** and **C**) show the proliferation of CCD-1079Sk cells and H9c2(2–1) cells after treatment with empty and 5-FU-loaded CD21chol. (**B** and **D**) show the viability of CCD-1079Sk cells and H9c2(2–1) cells after treatment with empty and 5-FU-loaded CD21chol. Data are presented as mean ± SD. Statistical analysis was performed using one-way ANOVA followed by Tukey’s post hoc test. Statistical significance was defined as follows: *p < 0.05, **p < 0.01, ***p < 0.001, ****p < 0.0001.

As illustrated in Figure 2, the evaluation assesses the impact of CD21chol and its complexes with 5-FU on the proliferation and viability of CCD-1079SK fibroblast cells and H9c2(2–1) cardiomyocyte cells. The study demonstrated that the empty CD21chol and the 5-FU-loaded CD21chol affected the metabolic activity and viability of the treated cells compared to untreated cells. In fibroblast cells, exposure to an empty CD21chol resulted in a 30% reduction in metabolic activity and survival. The presence of drug molecules in CD21chol carriers, regardless of the number of loaded molecules, has decreased fibroblast cell survival by 40–50%, indicating that these carriers are moderately cytotoxic.

The assessment of cardiomyocyte cell survival revealed a 30% reduction in viable cells compared to the control group. A comparable effect was observed in cells treated with empty carriers and carriers encapsulated with 5-FU, indicating that these compounds are weakly toxic, based on ISO 10993–5 standard criteria.^25^

**Figure 3:**
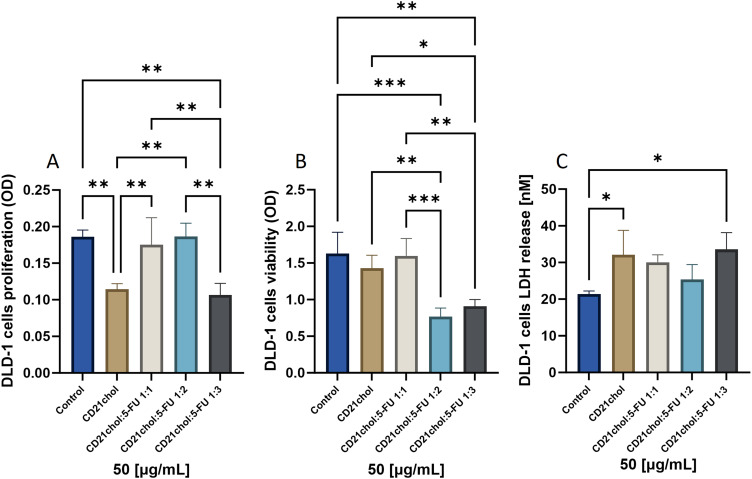
Evaluation of cytotoxicity of CD21chol and its complexes with 5-FU against colon cancer cells of DLD-1 lineage. (**A**–**C**) show the impact of empty and 5-FU-loaded CD21chol on proliferation, viability, and leakage of LDH via plasma membrane destruction, respectively. Data are presented as mean ± SD. Statistical analysis was performed using one-way ANOVA followed by Tukey’s post hoc test. Statistical significance was defined as follows: *p < 0.05, **p < 0.01, ***p < 0.001.

As depicted in Figure 3, the evaluation explores the impact of empty and 5-FU-loaded CD21chol on the proliferation and viability of the DLD-1 cell line. The results demonstrated that the tested compounds inhibited DLD-1 cell metabolic activity compared with untreated cells (Figure 3A). It was observed that the presence of two or three molecules of 5-FU significantly reduced the survival of DLD-1 cells by greater than 60% (Figure 3B). An extracellular LDH assay confirmed this observation. A statistically significant increase in LDH enzyme concentration was observed in the medium of cells treated with synthesized CD21chol (Figure 3C). The results obtained may suggest that the test molecules affect the permeability of the plasma membrane in treated cells by interacting with the surface cholesteryl groups of CD molecules and membrane phospholipids.

The findings from the MTT, neutral red, and LDH release assays suggest that the CD-based carrier for delivering 5-FU significantly decreased CRC survival. This effect was achieved by inducing cellular toxicity via multiple pathways, including inhibition of metabolic activity, plasma membrane damage, and extracellular LDH release.

### Assessment of Apoptotic Pathways via Caspase Activation

The primary goal of anticancer therapies is to induce the programmed death of malignant cells through apoptosis. The activation of the protease caspase is a prerequisite for the initiation of apoptosis. Caspase activity assays were used to quantify apoptosis, as caspases play a key role in both initiating cell death and modulating the organism’s inflammatory response. The extrinsic pathway of classical, caspase-dependent apoptosis is triggered by receptor binding, which recruits FADD- and TRADD-like adapter molecules (DAD) to the death domain. Procaspase-8 then binds to the death receptor domains of these adapters, activating it. Caspase-8 activates either more executive caspases (3 and 7) or the Bcl2 protein family, which moves to the mitochondria and induces the release of cytochrome C.^27^ The intrinsic pathway of caspase-dependent apoptosis occurs when cellular stresses, including heat shock, DNA damage, and oxidative stress, lead to the release of cytochrome c or Smac/DIABLO from the mitochondria to the cytosol. Then, the formation of an apoptosome (a caspase-activating complex) is triggered by cytochrome c, resulting in the activation of caspase-9, which in turn activates the subsequent effector caspases −3 and −7.^28^ Caspase-3 and −7 activation cause DNA fragmentation and cell death. These proteins are essential for the degradation of cellular components during the final stages of apoptosis. The actions of these proteins create a cascade that ensures the controlled removal of damaged cells, which is crucial for cancer therapy and for the response to cytotoxic agents.^28–30^

**Figure 4:**
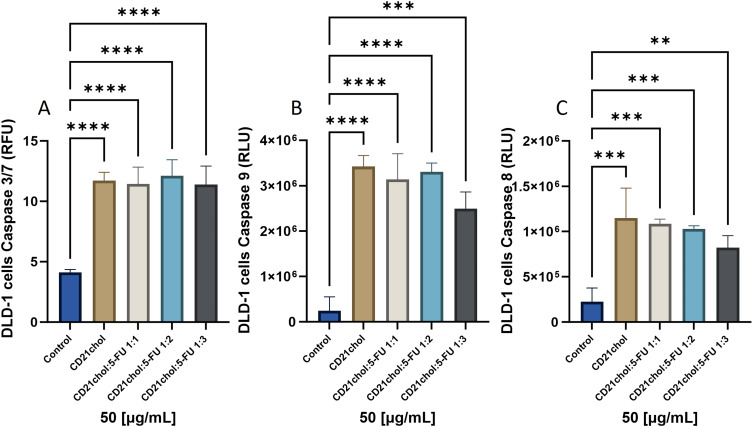
Assessment of proapoptotic caspase activity in DLD-1 cells under CD21chol treatment and its complexes with 5-FU. (**A**–**C**) indicated caspase 3/7, 9, and 8 activity in DLD-1 cells, respectively. Data are presented as mean ± SD. Statistical analysis was performed using one-way ANOVA followed by Tukey’s post hoc test. Statistical significance was defined as follows: **p < 0.01, ***p < 0.001, ****p < 0.0001.

In the subsequent phase of the study, the capacity of the examined CD-based carriers to induce apoptosis was determined. This study evaluated the activity of crucial proapoptotic caspase pathway proteins after a 24-hour incubation with the tested compounds. As illustrated in Figure 4A, the activation of caspase 3/7 in DLD-1 cells treated with modified CD21chol, empty, or 5-FU-loaded CD21chol showed a threefold increase compared to the untreated control group.

The results for caspase-9 activity in DLD-1 cells showed that incubation with CD21chol-based carriers led to a statistically significant increase in caspase-9 activity compared to untreated control cells. The aforementioned results suggest that the tested carriers effectively activate the mitochondrial apoptosis pathway (Figure 4B), which is also associated with their ability to reduce the cellular metabolic activity. A subsequent analysis of caspase-8 activity in the extrinsic pathway revealed a statistically significant increase in caspase-8 activity after treatment with CD21chol carriers compared with untreated cells.

## Discussion

Recent advancements highlight the multifunctionality of cyclodextrins (CDs) in contexts beyond traditional pharmaceutical applications. CDs have novel uses in nanomedicine, diagnostics, bioimaging, smart material engineering, and stimuli-responsive drug release systems.^31^ CDs are natural, macrocyclic oligosaccharides that can form inclusion complexes with various substances. One well-known natural polymer at the nanoscale is β-cyclodextrin (β-CD), which consists of seven α-D-glucose units linked by α-1,4-glucose bonds. The central cavity of β-CD is lipophilic, while the outer surface is hydrophilic, which can be modified during chemical synthesis. These properties enable β-CD to interact with lipophilic guest molecules and form host-guest inclusion complexes through self-assembly.^32^ It has also been reported that β-CD can influence the physicochemical and pharmaceutical properties of drugs, via improving stability and oral bioavailability.^33^ Furthermore, CDs are capable of providing sustained drug release through the formation of inclusion complexes, which may enhance the bioavailability and biocompatibility of the incorporated compounds. Furthermore, by encapsulating drug molecules within their cavity, CDs can improve chemical stability and increase aqueous solubility.^34^

Our recent findings showed that cholesteryl-substituted cyclodextrins form stable supramolecular assemblies, which may favour interactions with lipid membranes and, consequently, influence cytotoxic responses. The present findings indicate that the biological activity of the CD21chol:5-FU system is closely linked to the drug-to-carrier molar ratio and the physicochemical characteristics of the complexes formed. Importantly, the observed effects cannot be explained by a simple increase in drug content. Instead, they appear to result from the interplay between complex stability, membrane interactions, and the intracellular availability of 5-FU. This type of behaviour has been described previously for chemically cyclodextrin systems.^18^

In another study, stable 1:1 host–guest inclusion complexes of 5-FU with α-cyclodextrin (α-CD) and β-cyclodextrin (β-CD) were prepared. They assessed the complexes’ cytotoxicity against several cancer cell lines, including gastrointestinal (GI) and colon, liver, and stomach cancers, as well as breast and lung cancers. The authors observed divergent cytotoxic effects among the tested cells. Particularly, they observed increased anticancer effects in A-549 cells with the α-CD complex and in MCF-7 cells with the β-CD complex. It is interesting that the evaluation of the empty carrier’s cytotoxic activity also showed divergent cellular effects. For example, a marked cytotoxic effect was noted in MCF-7 cells after exposure to α- and β-CD at concentrations up to 431 and 352 µM, respectively. In turn, β-CD demonstrated an ability to inhibit the growth of GI malignancies; at concentrations of about 700 µM and 600 µM, respectively, the proliferation of HepG2 and Caco-2 cells was inhibited. The observed differences in cell lines’ cytotoxicity under treatment with empty and drug-loaded CD were attributed to differences in cell membrane composition, particularly cholesterol content.^15^ It has been suggested that cyclodextrins interact with membrane cholesterol, potentially extracting it from lipid rafts. The above-mentioned process can promote endocytosis or cause transient cell membrane damage, thereby facilitating the cellular uptake of drug-cyclodextrin complexes.^16^ Thus, the organization of the cell membrane and cholesterol levels determine how cancer cells respond to cyclodextrin treatment. Moreover, this molecular interaction might result in deregulation of membrane permeability, the release of cytoplasmic enzymes (eg, LDH), and the secondary activation of apoptotic pathways. Reports on cholesterol-containing polymers suggest that the cholesteryl moiety can significantly enhance the cellular uptake of drug carriers by binding to lipid rafts.^35^ This interaction may promote partial membrane destabilization and facilitate intracellular drug release, particularly in pathological cells. Cholesterol acts as a stabilizing and complexing part for hydrophobic drugs, improving their encapsulation and carrier stability.^36^ Accordingly, in the context of CD21chol, the cholesteryl units may perform a similar function by increasing interaction with membrane lipids and facilitating the transport and release of 5-FU, which could explain the observed increase in cytotoxicity toward cancer cells while maintaining relative safety for healthy cells. In essence, a considerably reduced dosage of the chemotherapeutic agent effectively inhibits cell proliferation, decreases viability, and induces apoptosis in the treated cells. The proposed mechanism of direct interaction between the CD21chol surface and cell membrane is consistent with the experimental findings. The carrier concentration used in the experiment was 4.5 µM, and the drug content was 0.013, 0.009, and 0.0045 µM at molar ratios of 1:3, 1:2, and 1:1, respectively. Specifically for the DLD-1 line, reported IC_5__0_ values are around 210 µM after 24 h and 380 µM after 48h (MTT assays), confirming the moderate sensitivity of this line to 5-FU.^37^,^38^ In the context of 5-FU cytotoxicity, the authors demonstrate that free 5-FU is highly toxic to healthy cells. The results indicated that CRL-1475 fibroblast cells exhibited ~5% viability at concentrations of 5 and 25 µg/mL. In contrast, H9c2(2–1) cardiomyocytes demonstrated greater resistance, with viability of ~45% at 5 µg/mL and ~40% at 25 µg/mL. These results further support the idea that combining 5-FU with CD21chol could mitigate toxicity in normal cells while maintaining or improving its effectiveness against cancer cells.^26^

The improvement in the antitumor activity of 5-FU after conjugation with β-cyclodextrin has also been previously described in the case of covalent 5-FU–β-CD conjugates. It has been demonstrated that such systems are capable of inhibiting tumor proliferation and growth more effectively in colorectal cancer models than the free drug. Furthermore, they have been shown to reduce toxicity to normal cells and limit liver and kidney damage in vivo. Furthermore, the conjugates exhibited prolonged half-life and a regulated release effect. This underscores the significance of the architecture of the drug-carrier system, encompassing the type of linker employed, in determining the biological response and therapeutic efficacy.^39^

It is well established that the physicochemical and morphological properties of drug carriers critically influence their therapeutic performance.^40^ In particular, changes in surface charge and ability to agglomerate might markedly affect the process of cellular internalization and, consequently, carrier efficiency. Novel β-cyclodextrin derivatives were obtained through functionalization with carboxymethyl and quaternary ammonium groups, yielding β-CD-based delivery systems for 5-FU.^41^ The cytotoxic activity of the resulting inclusion complexes was evaluated in HepG2 cancer cells. The results demonstrated that complexation of 5-FU with either carboxymethylated or quaternized β-CD did not significantly alter its antitumor efficacy. These findings confirm the suitability of β-CD derivatives as carriers for 5-FU. Importantly, the authors highlighted that nanoparticle surface charge plays a crucial role in cellular uptake, which may influence therapeutic performance. Moreover, the modified β-CD systems exhibited potential for sustained release and targeted drug delivery.^41^ As previously reported, β-CD complexation has been demonstrated to enhance the stability of drugs, modulate their biological activity, and reduce apparent toxicity, thereby confirming the potential of cyclodextrin-based systems in optimising the delivery of chemotherapeutic drugs. This finding supports our hypothesis that appropriate modification of cyclodextrin-based carriers allows for the optimisation of their biological properties and increased anticancer efficacy.^42^

In summary, the advantageous reduction of both therapeutic doses and associated systemic side effects has been previously studied and reported by other authors. It should be emphasized that our results complement these studies. Importantly, the activity of cholesterol-decorated β-CD, together with its unique physicochemical properties, offers the potential to improve the pharmacological performance of chemotherapeutic agents and to employ these carriers in controlled and personalized colorectal cancer therapy. It should be noted that the present study was conducted using a single colorectal cancer cell line (DLD-1), selected as a clinically relevant and widely used in vitro model. DLD-1 cells harbor key mutations commonly observed in colorectal cancer (eg, KRAS and BRCA2) and are tumorigenic, supporting their applicability in translational and xenograft-based studies.^43^ However, the genetic and phenotypic heterogeneity of cancer cells may influence cellular responses and limit the generalizability of the findings. Additionally, the simplified experimental conditions may not fully capture nanocarrier behavior under physiological settings. Further studies in more complex models, including in vivo systems, are therefore warranted.

## Conclusion

The observed shift in surface charge upon complexation, particularly the increased negative zeta potential for the 1:3 formulation, appears to enhance the biological activity of the CD21chol system. This complex exhibited the strongest cytotoxic effect against DLD-1 colon cancer cells, as evidenced by reduced proliferation, decreased metabolic activity, and significant activation of caspases 3/7, 8, and 9. These findings suggest that tuning the carrier’s surface charge through drug loading may improve its interaction with cellular membranes and thereby increase anticancer efficacy. The findings of this study demonstrate that modified CD21chol cyclodextrins exhibit cytotoxicity against colorectal cancer cells (DLD-1). The DLD-1 cell line was selected as a well-established and clinically relevant model of colorectal cancer. However, it should be noted that the use of a single cell line constitutes a limitation of this preliminary in vitro study and may affect the generalizability of the results. This phenomenon is attributed to the disruption of cell membrane integrity and the subsequent activation of the proapoptotic caspase pathway, encompassing the mitochondrial pathway. The study with non-tumorigenic cells indicates that CD21chol carriers, despite their notable efficacy against cancer cells, elicit moderate cellular toxicity against fibroblasts and cardiomyocytes. Consequently, further research and modification are necessary to modulate their activity and selectivity. In light of the findings, it can be concluded that the results obtained emphasize the potential of cyclodextrin-based carriers as a drug delivery system for treating colorectal cancer and for their application in anticancer therapy. Future studies should focus on evaluating interactions with biological components, including protein corona formation, as well as determining the localization of the system under more physiologically relevant conditions, to better understand it’s in vivo behavior and further optimize its therapeutic potential.
